# Diagnostic Performance of the Maximal Systolic Acceleration for Detecting a Significant Stenosis in the Aortoiliac and Popliteal Pathway: A Retrospective Cohort Study

**DOI:** 10.1177/15266028241309267

**Published:** 2024-12-26

**Authors:** Siem A. Willems, Saskia G. Dolfing, Rob C. van Wissen, Rutger W. van der Meer, Jan van Schaik, Joost R. van der Vorst, Abbey Schepers, Jaap F. Hamming, Jeroen J. W. M. Brouwers

**Affiliations:** 1 Department of Vascular Surgery, Leiden University Medical Center, Leiden, The Netherlands; 2 Department of Interventional Radiology, Leiden University Medical Center, Leiden, The Netherlands

**Keywords:** peripheral arterial disease, diagnostics, point-of-care testing, maximal systolic acceleration

## Abstract

**Introduction:**

Identifying peripheral arterial disease (PAD) remains challenging with currently used bedside tests. The maximal systolic acceleration (ACC_max_) is a promising noninvasive parameter measured by duplex ultrasonography and reflects the arterial perfusion proximal to its measurement point. The principal aim of this study was to analyze the diagnostic accuracy of the ACC_max_ for detecting significant stenosis in different arterial segments, which could be useful in clinical decision-making.

**Materials and Methods:**

A retrospective cohort study was conducted in a tertiary referral hospital. Patients aged 18 years and older who underwent ACC_max_ measurement(s) alongside computed tomography angiography (CTA) of the abdominal aorta and lower extremities were qualified for inclusion. A significant stenosis was defined as a lumen reduction of more than 50% on CTA. Diagnostic accuracy of the ACC_max_ was investigated for the aortoiliac and popliteal arterial pathways.

**Results:**

A total of 196 patients (373 limbs) were included in the study. Diagnostic performance of the ACC_max_ (cut-off value of 7.70 m/s^2^) to detect a significant stenosis in the aortoiliac pathway showed a sensitivity of 89%, specificity of 97%, positive likelihood ratio of 29.23 and negative likelihood ratio of 0.12 (area under the curve [AUC] 0.941). For the popliteal pathway (cut-off value of 6.30 m/s^2^), these results were 90%, 95%, 17.14 and 0.12, respectively, with an AUC of 0.958.

**Conclusion:**

The ACC_max_ showed a promising diagnostic accuracy for detecting a significant stenosis in the aortoiliac and popliteal pathway.

**Clinical Impact:**

The maximal systolic acceleration (ACC_max_) is a promising non-invasive parameter measured by duplex ultrasonography to diagnose peripheral arterial disease (PAD) and reflects the arterial perfusion proximal to its measurement point. This study focused on its diagnostic accuracy to detect a significant stenosis in the aortoiliac and popliteal pathway, which revealed to be promising with excellent sensitivity and specificity. These findings suggest that ACC_max_ measurements could play a key role in developing a new diagnostic approach for PAD.

## Introduction

Peripheral arterial disease (PAD) is a common condition characterized by progressive narrowing of arteries, leading toreduced blood flow to the limbs.^1^ Proximal disease (i.e. PAD of the aortoiliac and femoropopliteal arteries) accounts for approximately 75% of all atherosclerotic lesions.^2,3^ Proximal atherosclerosis is closely related to cigarette smoking, hypertension, and hypercholesterolemia, whereas below the knee involvement is predominantly associated with diabetes mellitus (DM).^3^

To diagnose PAD, different invasive and noninvasive tests are available. Currently, ankle-brachial index (ABI), toe-brachial index (TBI), and toe pressure (TP) are most frequently used as bedside tests worldwide.^4,5^ Although these tests can be useful in the diagnostic work-up of PAD, they do not provide information about the location of disease.^6^ In planning treatment, it is therefore necessary to perform additional testing, such as duplex ultrasonography (DUS), computed tomography angiography (CTA), magnetic resonance angiography (MRA), or digital subtraction angiography (DSA). While these reference tests are reliable, they are time-consuming, expensive, impose radiation, or can be nephrotoxic if intravenous contrast is used.^4^

The maximal systolic acceleration (ACC_max_) is a promising noninvasive parameter generated by DUS and reflects arterial perfusion at the level of measurement. So the ACC_max_ provides a representation of the arterial pathway proximal to the measurement point.^7^ Previous research showed excellent diagnostic performance of the ACC_max_ to diagnose and exclude PAD when measured in the distal anterior tibial artery (ATA) and distal posterior tibial artery (PTA).^8^ Maximal systolic acceleration measurements can be obtained at different arterial target points (eg, in the popliteal artery), giving more detailed information about the location of stenosis. This could be of great relevance in clinical decision-making, particularly if the test could exclude stenosis or occlusion in proximal limb segments (ie, aortoiliac and/or popliteal pathways). Such a diagnostic strategy could increase time-efficiency and reduce the need for additional imaging. In general, ACC_max_ measurements are relatively easy to obtain and take about 1 to 5 minutes for each target point.^7^

The primary aim of this study was to analyze the diagnostic accuracy of the ACC_max_ to detect a significant stenosis in the aortoiliac and popliteal pathways. Secondary objectives included the evaluation of the diagnostic performances of multiple ACC_max_ cut-off values to confirm or rule out a significant stenosis. A subgroup analysis was performed for patients with claudication symptoms.

## Materials and Methods

This retrospective diagnostic accuracy study was performed at a tertiary referral hospital. Ethical approval was obtained from the Research and Science Committee (2022-047) and granted on September 7, 2022. Data were extracted by using CT-cue and included patient information from January 1, 2008 until the December 31, 2022.

### Selection Criteria and Study Characteristics

Patients older than 18 years who underwent ACC_max_ measurement(s) of the (deep) femoral and/or popliteal artery alongside run-off CTA of the abdominal aorta and lower extremity were included. The maximum interval between the 2 diagnostic tests was 6 months, with no intervening vascular interventions. Patients were only included if ACC_max_ measurements were performed prior to the CTA and thereby obtained blindly to the reference test. The contralateral limb was analyzed as well in case ACC_max_, and CTA was performed.

In addition to diagnostic test results, demographic information including age, sex, comorbidities (DM, chronic kidney disease (CKD), hypertension, hypercholesterolaemia, cardiac disease (coronary artery disease, chronic heart failure and dysrhythmias), kidney transplantation and smoking) and Fontaine classification were collected from electronic patient files. Two researchers (SW and SD) extracted the data and compiled the database.

### Arterial Pathways

**Figure 1.**
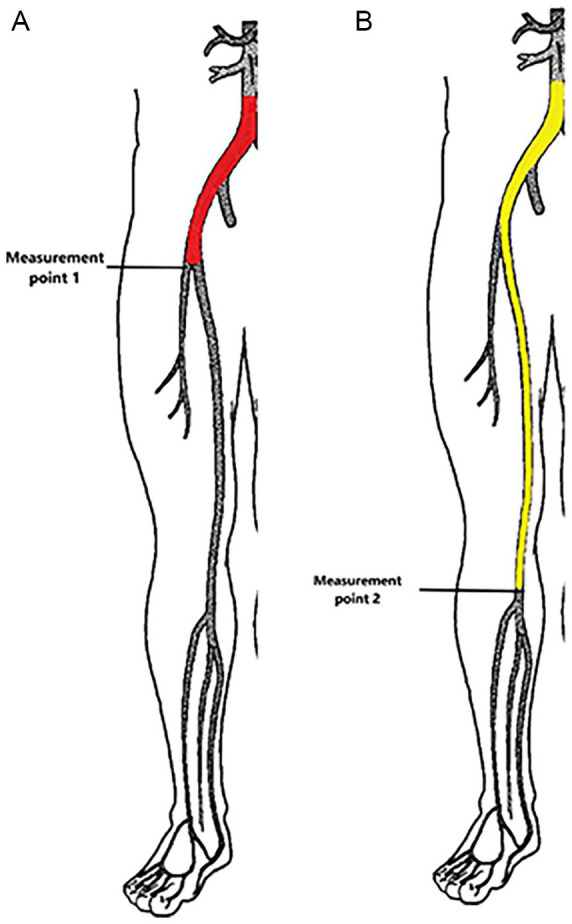
Visual overview of measurement points and corresponding arterial pathways. (A) Measurement point 1: Located at the proximal deep femoral artery. The aortoiliac pathway is highlighted by the red line and includes the distal abdominal aorta, common iliac artery, external iliac artery, and common femoral artery (CFA). (B) Measurement point 2: Located at the distal popliteal artery. The popliteal pathway is represented by the yellow line and includes the distal abdominal aorta, common iliac artery, external iliac artery, CFA, superficial femoral, and popliteal artery.

A significant stenosis was defined as >50% lumen reduction on CTA, which is consistent with previous systematic reviews.^9–11^ A contrast-enhanced CTA was used with spatial resolutions of at least 0.5 mm. To reliably assess diagnostic accuracy, arterial pathways were defined for the aortoiliac and popliteal pathways.^8^ This concept is derived from the target arterial pathway, which is used in the international guideline of chronic limb threatening ischemia.^5^ An arterial pathway was defined as all arterial segments proximal to the designated point of ACC_max_ measurement. The aortoiliac pathway included the distal abdominal aorta, common iliac artery, external iliac artery and common femoral artery (CFA). For the popliteal pathway, the distal abdominal aorta, common iliac artery, external iliac artery, CFA, superficial femoral, and popliteal artery were included.^8^ The ACC_max_ measurement point of the aortoiliac pathway was at the CFA or deep femoral artery proximally, whereas the ACC_max_ measurement of the popliteal pathway was obtained at the distal popliteal artery. Figure 1 shows an overview of these pathways.

### Maximal Systolic Acceleration

**Figure 2.**
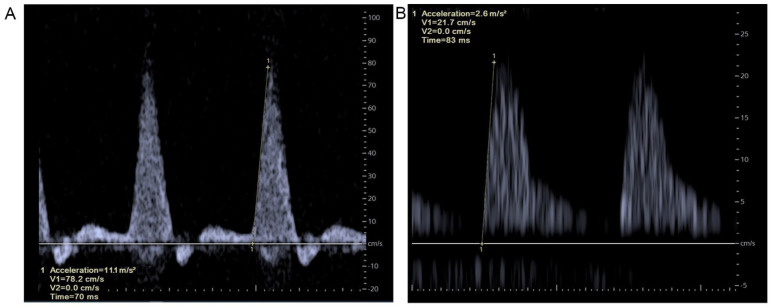
Doppler ultrasonography. (A) Normal triphasic waveform showing an ACC_max_ measurement of 11.1 m/s^2^, calculated at the maximal slope in the systolic phase of an arterial flow (tangent line indicated by 1). (B) Abnormal waveform showing an ACC_max_ measurement of 2.6 m/s^2^, calculated at the maximal slope in the systolic phase of an arterial flow (tangent line indicated by 1). Note. The differences in scales between the figures.

The ACC_max_ is a velocimetric doppler-derived parameter (with point-of-care DUS), which is measured in the systolic phase of an arterial flow (expressed in m/s^2^) and reflects arterial perfusion at the level of measurement. So, the ACC_max_ provides a representation of the arterial pathway proximal to the measurement point. As previously described by Brouwers et al^7^ and Willems et al,^8^ this parameter is generated during DUS and calculated in a representative waveform at the maximal slope of the upstroke. A tangent line can be made manually by adding 2 points exactly following this maximal slope (Figure 2). The acceleration of the tangent line is automatically calculated in m/s^2^ (ACC_max_). In general, a steeper curve represents a better hemodynamic (macrovascular) perfusion profile of the measured artery.^8^ One measurement takes about 1 to 5 minutes. An Acuson S2000 System (Siemens Medical Solutions, Ultrasound Division, Issaquah) equipped with a 9L4 9-4 MHz linear transducer was used during the entire study period. No additional software is required to obtain the ACC_max_. One vascular technician performed the ACC_max_ measurements per patient. The scale (velocity and time) and sweep were optimized for each examination. The velocity scale was set slightly higher than the peak systolic velocity (Doppler waveform covering at least three fourth of the window is necessary). The time scale contained a maximum of 3 to 4 heartbeats. In case dysrhythmias were present, the most representative waveform for that particular patient was used. Previous studies showed a high interobserver agreement for ACC_max_ measurements (intra-class coefficient 0.97–0.99) in an experimental setting.^7,12^ Moreover, a strong correlation between the severity of stenosis and ACC_max_ values was found in these studies.

### Statistical Analysis

Descriptive statistics were calculated for participant demographics using means for continuous variables and proportions for categorical variables. Diagnostic accuracy was measured by calculating sensitivity, specificity, positive likelihood ratio (PLR) and negative likelihood ratio (NLR). Positive likelihood ratio and NLR are derivatives of sensitivity and specificity and represent the effect on posttest probability of disease. The ratios were interpreted as follows; no effect (PLR 1, NLR 1), small effect (PLR 2–5, NLR 0.2–0.5), moderate effect (PLR 5–10, NLR 0.1–0.2) and large effect (PLR >10, NLR <0.1).^13^ Analyses were made for each pathway separately.

Youden’s Index was used to determine optimal cut-off values of the ACC_max_ to detect a significant stenosis for each pathway. In addition, diagnostic performance was calculated for all cut-off values from 4 to 12 m/s^2^. Receiver operating characteristics (ROC) curve analyses were performed to estimate the area under the curve (AUC) with 95% confidence intervals (CIs). A subgroup analysis was performed for patients with claudication symptoms (identified as Fontaine IIA or IIB). Calculations were executed in SPSS for Windows version 25.0 (IBM, Armonk, NY: IBM Corp).

## Results

A total of 196 patients (373 limbs) were included in this study. Mean age of patients was 68 years old and most of the patients were male (62%). Most were known with hypertension (68%), history of smoking (67%), and hypercholesterolaemia (60%). Diabetes mellitus was present in 44% of cases and approximately one third of patients suffered from CKD (31%). All patient characteristics can be found in Table 1.

**Table 1.** Patient characteristics.

Variable	N (%)
Total patients and limbs	196–373 (100)
Sex	
Male	122 (62.2)
Female	74 (37.8)
Side	
Right limb	198 (53.1)
Left limb	175 (46.9)
Mean age, years (SD)	68.6 (10.3)
Age range, years	42–91
Comorbidities	
Diabetes mellitus	87 (44.4)
Chronic kidney disease (eGFR < 60 mL/min/1.73 m^2^)	61 (31.1)
Cardiac disease	90 (45.9)
Hypertension	134 (68.4)
Hypercholesterolaemia	118 (60.2)
Kidney Transplantation	16 (8.2)
History of smoking	131 (66.8)
Post-intervention in medical history	
Yes (PTA, stent, bypass, EVAR)	48 (24.5)
Fontaine Classification	
Fontaine IIa	52 (13.9)
Fontaine IIb	118 (31.6)
Fontaine III	66 (17.7)
Fontaine IV	112 (30.0)
Asymptomatic	18 (4.8)
Missing	7 (1.9)

Abbreviations: EVAR, endovascular aortic repair; PTA, percutaneous transluminal angioplasty; SD; standard deviation.

### Diagnostic Accuracy Aortoiliac and Popliteal Pathway

Table 2 presents the diagnostic performance of the ACC_max_ to diagnose a significant stenosis in both the aortoiliac and popliteal pathway according to highest Youden Index.

**Table 2.** Diagnostic Accuracy of Maximal Systolic Acceleration Per Pathway.

	Cut-off value ACC_max_ according to Youden Index	AUC (95% CI)	Sensitivity % (95% CI)	Specificity % (95% CI)	PLR (95% CI)	NLR (95% CI)
Aortoiliac pathway (n = 171)	7.70	0.941 (0.903–0.979)	89 (80.9–94.0)	97 (89.5–99.6)	29.2 (7.5–114.6)	0.12 (0.07–0.20)
Popliteal pathway (n = 354)	6.30	0.958 (0.939–0.977)	90 (86.3–93.4)	95 (85.4–98.9)	17.1 (5.7–51.6)	0.10 (0.07–0.15)

Abbreviations: AUC, area under the curve; CI, confidence interval; NLR, negative likelihood ratio; PLR, positive likelihood ratio.

**Figure 3.**
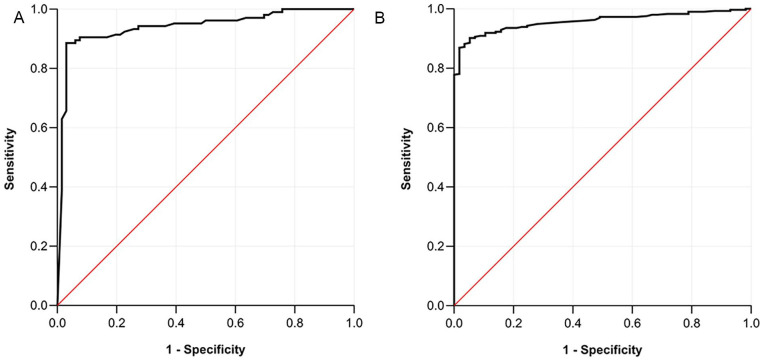
Receiver operating characteristics curve for maximal systolic acceleration versus arterial pathway per pathway. (A) aortoiliac pathway and (B) popliteal pathway.

For the aortoiliac pathway (n = 171), an optimal ACC_max_ cut-off value of 7.70 m/s^2^ was found with an AUC of 0.941 (CI 0.903–0.979). Corresponding sensitivity and specificity were 89% and 97%, respectively, with a PLR of 29.23 and an NLR of 0.12. Figure 3A displays the ROC-curve for the aortoiliac pathway.

For the popliteal pathway (n = 354), an optimal ACC_max_ cut-off value of 6.30 m/s^2^ was identified with an AUC of 0.958 (CI 0.939–0.977). Diagnostic performance included a sensitivity of 90% and specificity of 95%, leading to a PLR of 17.14 and NLR of 0.10. Figure 3B shows the ROC-curve for the popliteal pathway.

### Cut-Off Values

The diagnostic accuracy of the ACC_max_ for multiple cut-off values from 4 to 12 m/s^2^ is depicted in Table 3. As the ACC_max_ is (artificially) set higher, sensitivity increases and specificity decreases. The PLR and NLR follow the same trend, indicating that a higher ACC_max_ leads to a better performance in ruling out disease.

**Table 3.** Diagnostic Accuracy of Maximal Systolic Acceleration for Different Cut-Off Values Per Artery.

	Cut-off values	Sensitivity (%)	Specificity (%)	PLR	NLR
Aortoiliac pathway	4 5 6 7 8 9 10 11 12	80 84 87 88 89 90 91 93 95	97 97 97 97 94 92 87 74 60	26.4 28.0 28.6 28.9 14.6 11.9 6.6 3.6 2.4	0.21 0.16 0.14 0.13 0.12 0.10 0.11 0.09 0.08
Popliteal pathway	4 5 6 7 8 9 10 11 12	83 88 90 91 92 93 95 97 98	98 96 95 91 88 84 72 44 38	47.4 25.1 17.1 10.4 7.5 5.9 3.4 1.7 1.4	0.17 0.13 0.11 0.10 0.09 0.08 0.07 0.06 0.06

Abbreviations: NLR, negative likelihood ratio; PLR, positive likelihood ratio.

### Claudication Symptoms

In total, 170 limbs with Fontaine IIA or IIB were identified. Within this group, 58% were male and a higher percentage of smoking (80%) and hypercholesterolaemia (74%) was present. For the aortoiliac pathway (n = 87), an optimal cut-off value of 7.90 m/s^2^ with an AUC of 0.938 (CI 0.873–1.000) was found for detecting a significant stenosis. This led to a sensitivity and specificity of 91% and 93%, respectively, with corresponding positive and NLRs of 13.68 and 0.09. For the popliteal pathway (n = 158), ROC-curve analysis showed an AUC of 0.964 (CI 0.936–0.993) with a cut-off value of 7.00 m/s^2^. Sensitivity and specificity were 93% and 92%, with a PLR and NLR of 11.10 and 0.08, respectively. At a cut-off value of 9 m/s^2^, an NLR of 0.05 was obtained.

## Discussion

The results of this study show that ACC_max_ could have important additional value in the diagnostic work-up of patients with PAD. The ACC_max_ measurements in the proximal femoral artery and distal popliteal artery are both effective in detecting and ruling out significant stenosis proximal to the measurement point (aortoiliac and popliteal arterial pathways, respectively, as explained in Figure 1). Incorporating these specific ACC_max_ measurements in a diagnostic work-up would provide a direct overview of the location of stenosis, which could be helpful in clinical decision-making.

The ACC_max_ measurements of distal ATA and PTA have previously shown to be an accurate point of care test to diagnose and rule out PAD. Its diagnostic accuracy was promising compared to current regular bedside tests and did not diminish in patients prone to medial arterial calcification.^8^ This study focused on a new diagnostic work-up for patients with PAD using ACC_max_ as noninvasive parameter, which showed an excellent diagnostic accuracy (AUCs of 0.94 and 0.96 for aortoiliac and popliteal pathway lesions, respectively). These results also showed that it is possible to exclude a stenosis proximal to the measurement point with a reasonable degree of certainty (NLRs of 0.12 and 0.10 for both pathways). This could be relevant in daily clinical practice, since the ACC_max_ can accurately exclude a stenosis proximal to the popliteal artery with a simple measurement.

By combining this study with a previously published study, a new noninvasive diagnostic work-up strategy can be introduced.^8^ In case a patient presents with a suspicion of PAD, ACC_max_ measurements of the ATA and PTA will be performed. If one of these measurements is below 5.5 m/s^2^, PAD is confirmed,^8^ and an ACC_max_ measurement should be obtained at the level of the distal popliteal artery and the deep femoral artery. By directly adding these 2 measurements, it is possible to further distinguish between the anatomical location of disease. If the ACC_max_ of the distal popliteal artery reveals to be normal (ACC_max_ > 6.30 m/s^2^), a significant stenosis in the iliac, femoral, or popliteal artery is very unlikely and crural pathology is confirmed. On the contrary, a result below 6.30 m/s^2^ in the popliteal artery indicates that more proximal disease could be present (as well). In that case, an ACC_max_ measurement of the deep femoral artery is very helpful in localizing most proximal disease. Supposing that the ACC_max_ in the deep femoral artery is normal (ACC_max_ > 7.70 m/s^2^), no relevant aortoiliac disease is present, and the most probable location of (first relevant) stenosis will be in the superficial femoral artery. If the ACC_max_ in the CFA is below 7.70 m/s^2^, the most proximal lesion should be expected in the aortoiliac pathway. This framework of measuring the ACC_max_ from distal to proximal could provide various advantages in different patient groups and would offer potential chances in diagnostic strategies. In total, such a work-up would take 15 to 20 minutes approximately. The following scenarios could be seen as new hypotheses and as a stepping stone to the development of such a diagnostic work-up.

The first group would consist of patients with diabetic foot ulcers (DFUs). These patients typically have most of their atherosclerotic lesions in crural vessels, which is difficult to assess with CTA. In these cases, the same diagnostic work-up as described before could be used. For example, if the ACC_max_ is 1.0 m/s^2^ in the ATA, 1.5 m/s^2^ in the PTA and normal in the popliteal artery (ie, 12.5 m/s^2^), the presence of solely crural pathology has been confirmed. In such a case, one may choose to perform directly an antegrade DSA (including an endovascular crural intervention if possible) without additional imaging, as conventional DUS/CTA/MRA is often insufficient to make an accurate endovascular crural treatment strategy. This diagnostic work-up is time-saving and holds significant promise for hospital logistics, as it reduces the need for numerous DUS/CTA/MRA in specific patient groups.

The second potential group would be patient suffering from claudication symptoms. In case a low ACC_max_ in the ATA and a high ACC_max_ in the popliteal artery is measured, PAD of crural origin is diagnosed. In patients with merely claudication symptoms, a crural intervention is usually not appropriate.^4^ Immediate referral for supervised exercise therapy could be provided. When following this work-up, no additional imaging modality (DUS/CTA/DSA) would be necessary. The subgroup analysis of patients with claudication symptoms in this study supported this strategy with an excellent diagnostic accuracy of the popliteal region (AUC of 0.964—sensitivity 93% and NLR 0.08 at a cut-off value of 7 m/s^2^).

From both examples, it can be deducted that ACC_max_ measurements as primary screening tool for PAD could lead to an optimization of hospital capacity planning. In both patient groups, less additional imaging would be necessary to provide an adequate therapeutic strategy. Next to better operational management with cost reduction, medical benefits are present as well. Particularly if less CTA-scans are performed, less radiation and nephrotoxicity will be imposed on patients. Another potential advantage would be that the actual measured ACC_max_ can be linked to a certain diagnostic performance. For instance, an ACC_max_ measurement of 11 m/s^2^ in the popliteal artery reflects an excellent NLR of 0.06, and could therefore accurately rule out a significant stenosis proximal to the measuring point. Table 3 shows the corresponding performances of several ACC_max_ values which are clinically useful.

Therefore, ACC_max_ measurements could have additional value in comparison with currently used bedside tests to diagnose or exclude PAD. The diagnostic accuracy of simple waveform analysis (at ankle region) to confirm PAD has been performed in multiple studies before and also evaluated in several systematic reviews.^9–11^ These reviews showed that monophasic or triphasic signals could help to diagnose or exclude PAD, but the interpretation of biphasic signals remains unclear, and distinguishing between mono-/biphasic and bi-/triphasic can be challenging due to the absence of clear thresholds. The ACC_max_ addresses this issue by providing a quantitative measure, which could be particularly important in cases with difficult or inaccurate ABI measurements, such as in diabetic or dialysis patients.

Although this diagnostic work-up strategy yields less anatomical information compared to CTA and MRA, crucial information can also be obtained directly with DUS (such as calcified spots in the CFA or target peak systolic velocity measurements of doubtful significant stenosis) since the ACC_max_ is also measured by DUS. Moreover, CTA would still be part of diagnostic strategies in patients with PAD, such as CFA and superficial femoral artery lesions, multilevel disease or in case more anatomical information is needed. Only in certain cases, as described in the discussion before, additional imaging could be left out.

### Limitations

The findings of this study have to be seen in light of some limitations. First, due to the nature of a retrospective design, selection bias may have been introduced. Second, since there was no prespecified protocol, not all limbs had the same ACC_max_ measurement points. This could have led to indication bias. Furthermore, in this study, we have combined the ACC_max_ measurements of the common and deep femoral artery as measurement point for the aortoiliac pathway. We would recommend to measure the ACC_max_ in the deep femoral artery, since this provides additional information about the patency of the CFA. At last, future studies examining the ACC_max_ should also focus on the effect of cardiac disease (such as a low left ventricular output and dysrhythmias) on measurements at different segments/levels.

## Conclusion

The ACC_max_ is a Doppler-derived parameter that exhibits a promising diagnostic accuracy in excluding significant stenosis in the arterial pathway proximal to its measuring point. We suggest adding 2 new ACC_max_ measurement points (deep femoral artery and distal popliteal artery) in the diagnostic work-up of patients with PAD. This would provide an immediate overview of the location of stenosis, which could be helpful in treatment decisions. In addition, this new diagnostic work-up is time-saving, noninvasive, and can result in fewer DUS/CTA/MRA scans for specific patient groups.
